# Improvement of Dietary Habits among German Medical Students by Attending a Nationwide Online Lecture Series on Nutrition and Planetary Health (*“Eat This!”*)

**DOI:** 10.3390/nu15030580

**Published:** 2023-01-22

**Authors:** Anna Helbach, Moritz Dumm, Katharina Moll, Tim Böttrich, Can Gero Leineweber, Wiebke Mueller, Jan Matthes, Maria Cristina Polidori

**Affiliations:** 1 Department II of Internal Medicine, Faculty of Medicine and University Hospital Cologne, University of Cologne, 50931 Cologne, Germany; 2 Institute of General Practice, Faculty of Medicine and University Hospital Cologne, University of Cologne, 50931 Cologne, Germany; 3 Center of Pharmacology, Faculty of Medicine, University Hospital of Cologne, University of Cologne, 50931 Cologne, Germany; 4 Faculty of Medicine, University Clinic Hamburg Eppendorf, 20251 Hamburg, Germany; 5 Faculty of Medicine, University of Gießen, 35392 Gießen, Germany; 6 Medical Department B of Internal Medicine, Brandenburg Medical School, University Hospital Ruppin-Brandenburg, 16816 Neuruppin, Germany; 7 Institute of Medical Statistics and Computational Biology, Faculty of Medicine, University Hospital Cologne, University of Cologne, 50931 Cologne, Germany; 8 Ageing Clinical Research, Department II of Internal Medicine and Center for Molecular Medicine Cologne, Faculty of Medicine and University Hospital Cologne, University of Cologne, 50931 Cologne, Germany; 9 Cologne Excellence Cluster on Cellular Stress-Responses in Aging-Associated Diseases (CECAD), University of Cologne, 50931 Cologne, Germany

**Keywords:** diet quality, dietary guidelines, nutrition surveys, medical students, behavioral change, medical training, nutritional medicine, planetary health

## Abstract

Nutrition is a major influential factor in optimizing human health and environmental sustainability. Medical students often do not follow national dietary guideline recommendations. Raising awareness of a healthy lifestyle is important as physicians with healthy lifestyle behaviors are more likely to counsel on nutrition. Our study aims to evaluate a Germany-wide online lecture series on nutritional medicine, *“Eat This!”*. Before and after the course, 520 medical students who participated and 64 who did not participate in the course (comparison group) filled out an online survey. To assess the students’ dietary habits, a validated FFQ was used. According to this questionnaire, only 31% of the lecture participants consumed enough fruits and 24% consumed enough vegetables, while almost half of the students exceeded the recommended maximum amount of crisps and sweets. After attending the lecture series, guideline adherence with respect to fruits and vegetables showed a significant increase, as did awareness of healthy nutrition and percentage of students with low-risk lifestyle habits. Our results show that low-threshold approaches, such as *“Eat This!”*, can positively influence the dietary behaviors and lifestyle habits of medical students. This can help future doctors fulfill their role in the fight against the global burden of non-communicable diseases.

## 1. Introduction

The EAT-Lancet Commission rates nutrition as a major influential factor in optimizing human health and, at the same time, the environmental sustainability of our modern lives [1].

On the one hand, nutrition has a major impact on the development of non-communicable diseases (NCDs), such as cancer, cardiovascular diseases, and diabetes [2]. On the other hand, global food systems are responsible for one third of global greenhouse gas emissions and fail to meet the food demands of the world’s growing population [3]. Last but not least, modern diets often result in the consumption of excess calories. Thus, current food systems are neither healthy nor sustainable [1].

Diet is considered healthy when it improves or maintains personal health, prevents diseases, and meets individual nutritional needs [4,5]. Fruits, vegetables, nuts, and whole grain products should make up the main dietary components. High consumption of red and processed meat is increasingly associated with all-cause mortality, whereas consumption of vegetables, fruits, fish, and nuts is associated with lower all-cause mortality [6]. The occurrence of NCDs is further dependent on lifestyle factors, such as smoking, alcohol abuse, physical inactivity, and body mass index (BMI) [7]. In 2017, NCDs accounted for 91% of annual deaths and 88% of all disability-adjusted life years (DALYs) in Western Europe [8]. Imbalanced diets were responsible for 9.6 million deaths worldwide in 2018 [9]. The prevention and treatment of NCDs contribute to approximately one third of total health care spending in industrial countries [10].

Physicians play a key role in combating NCDs. The American Heart Association recommends addressing nutrition and lifestyle topics more frequently during counseling [11]. To better combat this issue, nutrition and lifestyle topics should already be addressed in medical school. 

Personal lifestyle and dietary behaviors among medical students have already been studied in some countries to date. It has been shown that students often do not conform to national dietary guideline recommendations [12,13,14,15,16,17,18,19,20]. It has been demonstrated that a course on preventive medicine and nutritional medicine can lead to improvements in individual dietary habits [12,21].

Raising awareness of a healthy personal lifestyle and dietary behaviors among medical students is relevant, as there is an association between the frequency of lifestyle and nutritional counseling and physicians’ individual healthy lifestyle behaviors [22,23]. Additionally, a healthy lifestyle forms an important foundation for improving academic performance in medical school [24].

In Germany, there is a lack of data on the lifestyle behaviors of medical students and the impact of attending online courses about nutritional medicine on individual dietary behaviors and lifestyle. Our study aimed to close this knowledge gap by assessing medical students’ individual habits and awareness of healthy eating and demonstrating the impact on these parameters after the students participated in the nationwide online lecture series, *“Eat This!”*, on nutrition and planetary health.

## 2. Materials and Methods

### 2.1. Lecture Series

The lecture series *“Eat This!”* (German: *“Iss Das!”*) for medical students in Germany took place online from October 2020 to February 2021. It was implemented by a team of medical students and physicians with the support from a non-profit organization, the Physicians Association for Nutrition international (PAN int.), as a further development of a project that started in Cologne in 2020 [25]. Participation in the lecture series was voluntary but could be credited as an elective in the medical faculties of Cologne, Aachen, Giessen, and Düsseldorf. The series contents were adapted to German guidelines as published in the *Manual of Nutritional Therapy in Patient Care* [26]. The lectures included basic knowledge on nutritional medicine, public health, and planetary health. Relevant climate aspects of the Eat Lancet Commission and the UN sustainable development goals were also included [1,27]. The specific content of each lecture can be found in Table S1. The lecture series consisted of 11 lectures of two hours each, which took place live online via the video conference tool “Zoom” at weekly intervals. The lecture series comprised a total contact time of 22 h. At each lecture, presentations by two or three lecturers were followed by a discussion and a Q&A session moderated by the organizers. Qualified lecturers from the fields of medicine, psychology, pharmaceutics, nutritional science, and public health were selected according to their expertise in the respective topics of the lectures. This was determined by literature research and by the lectures already given at the universities.

### 2.2. Study Design and Participants

We analyzed the impact of participation in the lecture series on students’ individual nutritional habits in an observational prospective study with a pre–post analysis. No sample size was calculated in advance due to the difficulty in estimating the number of lecture participants in advance. Furthermore, participation was completely voluntary, the nationwide format was a novelty, and we did not want to limit the number of participants. Ultimately, 4000 medical students registered for participation in the lecture series.

Of the total 1531 study participants, 1224 lecture participants and 122 students from the comparison group constituted the cross-sectional cohort at baseline. For the purpose of further elucidating the practical significance of the lecture series, a group of students not participating in the course was assessed. In order to recruit those students, the medical student associations of several German faculties were informed and asked to forward the invitation to complete the online survey to all medical students who did not plan on attending any of the lectures. The term chosen for this group was a “comparison group”.

The study participants (both groups) were invited to complete a questionnaire provided online through LimeSurvey prior to the start (November 2020) and after the conclusion of the lecture series (January 2021). As an incentive to participate in the study, 3 tickets for the VegMed congress and 10 vouchers for an organic supermarket were raffled among the participants.

**Figure 1:**
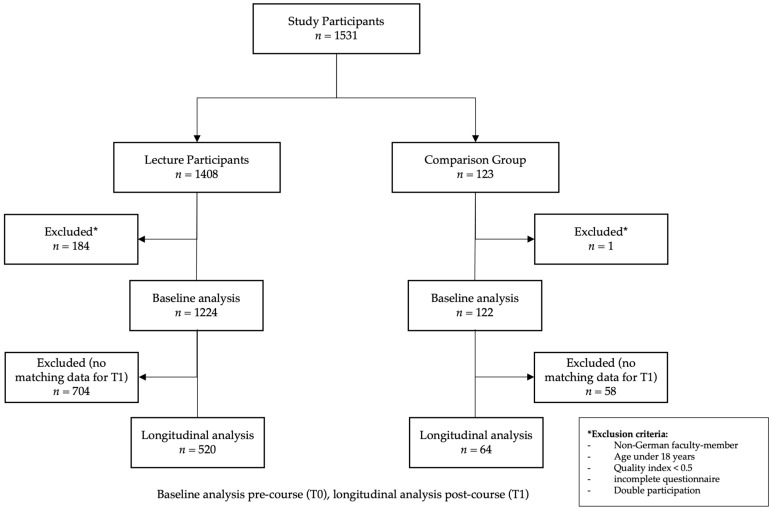
Flowchart of study participants. * *p* < 0.05.

Participation in the study was completely voluntary. The pseudonymization of the data and a data backup in accordance with the data protection guidelines were ensured. On the basis of the pseudonymization by means of a 5-digit code chosen by the participants themselves, it was possible to allocate the data records of each participant from T0 to T1 in order to carry out the longitudinal analyses. All students in the comparison group had to confirm that they had not attended any of the lectures prior to completing the questionnaire in order to avoid cross-contamination between students who had attended the lecture series and those who had not. Five hundred and twenty lecture participants and 64 medical students of the comparison group were included in the longitudinal analyses. Data were excluded (*n* = 184 for lecture participants, *n* = 1 for comparison group) when the participants belonged to non-German faculties, were under 18 years old, their quality index was <0.5 (quality index = participants interview time/average interview time), or the questionnaires were incomplete (Figure 1).

### 2.3. Questionnaire Development and Application

With respect to the current study, the survey contained questions about medical students’ dietary behaviors and lifestyle, awareness of healthy eating, and attitudes toward the impact of individual behavioral changes on the climate crisis. The items from a validated food frequency questionnaire (FFQ) on healthy diet were included, as well as the items that allowed a comparison to the recommendations of the German Nutrition Society (see below).

The questionnaire was completed online before (T0) and after (T1) the lecture series in order to assess any changes that could be attributed to the lecture series.

### 2.4. Dietary Quality Score

To assess the differences in the overall dietary quality of the participants before and after the lecture series, a dietary quality score (DQS) was calculated using the validated short-form food frequency questionnaire and DQS calculator by Cleghorn et al. [28]. As we conducted our study in Germany, we translated the underlying FFQ into German and referred to the recommendations of the German Nutrition Society [29]. Scores from 5 indicating a low dietary quality to 15 indicating a high dietary quality could be reached.

### 2.5. Students’ Adherence with the Recommendations of the German Nutrition Society

In addition to the FFQ by Cleghorn et al., information on the consumption of nuts, water, milk, and alcoholic drinks was asked to evaluate adherence to the German dietary guidelines [29]; the questions were asked separately for ten individual food groups. As suggested by other studies [30], the consumption frequencies given by the students were converted using standard portion sizes so that they reflected the amount consumed in grams per day or per week. The individual food items were then combined into 10 food groups and scores of 0 (below recommendations), 1 (meeting recommendations), or 2 (above recommendations) were given based on the recommendations of the German Nutrition Society (Supplementary Tables S2–S4).

### 2.6. Lifestyle

To measure the students’ lifestyle, alcohol consumption, physical activity, smoking habits, BMI, and diet quality were assessed to estimate a low-risk lifestyle score (Healthy Lifestyle Index, HLI), as suggested by Li et al. in 2019 [31].

A healthy lifestyle was, thus, defined as being physically active for at least 150 min per week [32], reporting low to no alcohol consumption [33], being a non-smoker, having a BMI between 18.5 and 24.9 kg/m^2^, and having healthy dietary habits [34]. Alcohol consumption was divided into 3 categories: low consumption (less than once per week), medium consumption (1–3 times per week), and high consumption (more than 4 times per week). Smoking habits were grouped into currently being a non-smoker or a smoker. The BMI was calculated by using self-reported information on height and weight.

Adhering to a healthy lifestyle was defined by having a DQS over 11 (thus being in the top 40% of participant distribution as suggested by Li et al. in 2019 [31]); engaging in physical activity at least 3 times per week; having a BMI between 18.5 and 24.9 kg/m^2^; alcohol consumption max. once per week; and no smoking. The lifestyle variables were then dichotomized into two categories to score as having low-risk lifestyle habit with the score of 1 and having a high-risk lifestyle habit with a score of 0. An overall score was calculated from the scores of these individual lifestyle variables. The higher the total score, the better the lifestyle. Students having scores of 4 or 5 of low-risk lifestyle habits were included in the variable “HLI ≥ 4”, as proposed by Richter et al. in 2021 [35].

### 2.7. Awareness of Healthy Eating and Planetary Health

To measure the awareness of healthy eating, the items “Healthy eating is important to me”, “I know which foods keep me fit and healthy”, “I know which foods keep me fit and healthy and eat accordingly”, and “There is a strong connection between healthy eating and individual well-being” were combined into the variable “Healthy Food Awareness”. The items “To limit the progression of the climate crisis, my diet should be mostly plant-based” and “To limit the progression of the climate crisis, I should consciously make sure to buy regional and local products” formed the variable “Personal Responsibility” with respect to the issue of planetary health. Awareness was assessed using a 5-point Likert scale ranging from −2 (totally disagree or very low) to +2 (totally agree or very high).

### 2.8. Statistical Analysis

The qualitative variables were expressed as frequencies and percentages. The quantitative variables were expressed as means and standard deviations or medians and interquartile ranges. The data were non-normally distributed since the Shapiro–Wilk test was <0.05. To primarily test for significant differences between pre- and post-course within each group, a Wilcoxon signed-rank test for continuous non-normally distributed data and a McNemar’s test for binary variables were performed. Secondly, for the comparison between the independent groups (lecture participants vs. comparison group) and variables, either a Mann–Whitney U test for continuous non-normally distributed (Shapiro–Wilk test < 0.05) variables or a χ^2^ test for categorical variables was used. If total cell counts were less than five, a Fisher’s exact test was used. For descriptive statistics and multivariate regression analysis, the variables Healthy Food Awareness and Personal Responsibility were dichotomized. Scores ≥ 1.5 were defined as high, while scores < 1.5 were defined as low awareness. A multivariate linear regression analysis was computed for all lecture participants at baseline to show the associations between the DQS (dependent variable) and the independent covariates, including age, gender, BMI, alcohol consumption, physical activity, smoking, high awareness of healthy eating, personal responsibility, study phase, and dietary practices. The goodness-of-fit of the model was assessed by using R^2^. All analyses were performed using IBM SPSS Statistics (SPSS Inc., Chicago, IL, USA, Version 27.0), and the graphs were designed using Graphpad Prism version 8.0.0. A *p* < 0.05 was considered statistically significant.

Participation in the study was voluntary and the participants signed the informed consent prior to completing the pseudonymized questionnaires. Ethics approval was obtained from the Ethics Committee of the University of Cologne: 20-1522_1.

## 3. Results

At pre-course, the lecture participants had a median age of 23 years old, were predominantly female (80%), and were mainly in their clinical study phase (74%). A total of 14.8% of lecture participants considered their diet to be vegetarian and 12.7% indicated vegan. There were no significant demographic differences between the lecture participants and the comparison group, nor between the participants in the cross-sectional and longitudinal analyses (Table 1).

### 3.1. Participants’ Dietary Quality Was Affected by Participation in the Lecture Series

**Figure 2:**
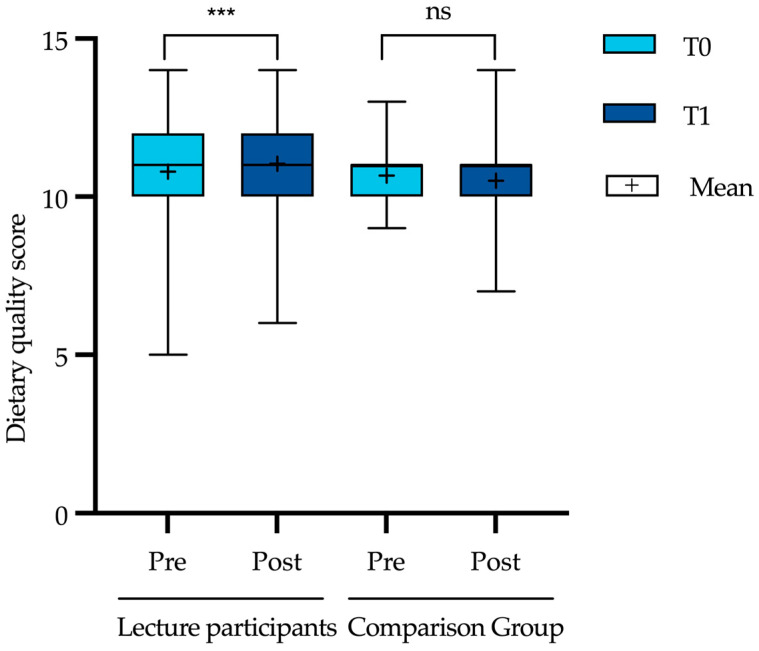
Mean dietary quality score of the lecture participants and the comparison group at pre-course (T0) and post-course (T1). Level of significance of observed differences between pre- to post-course as assessed by Wilcoxon signed-rank test, respectively: ns = not significant, *p* > 0.05; *** *p* < 0.001. Whiskers go from min. to max.

Before and after attending the lecture series, the students were asked to answer a validated questionnaire (FFQ), which was used to calculate a dietary quality score (DQS). The mean DQS of the lecture participants and the comparison group did not differ significantly at baseline (T0) (Table 1). At T1, however, a slight but statistically significant improvement in the DQS (10.8 ± 1.4 to 11.0 ± 1.3, *p* < 0.001) was observed in the group of lecture participants but not in the comparison group (10.67 ± 1.04 to 10.5 ± 1.2, *p* = 0.255) (Figure 2).

### 3.2. Students’ Adherence to the Recommendations of the German Nutrition Society

**Figure 3:**
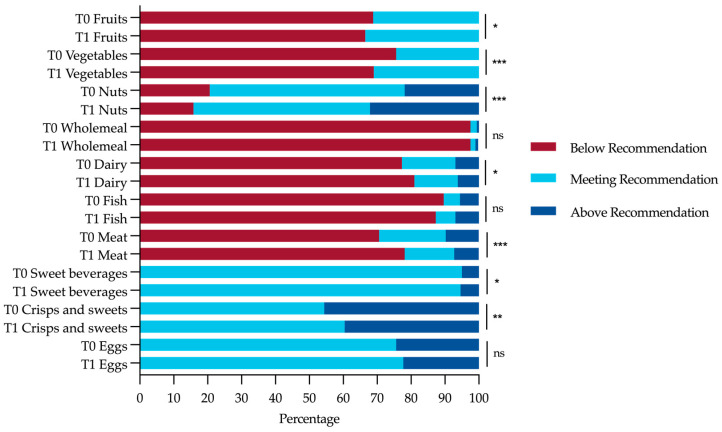
Percentage of the lecture participants’ food intake in relation to the national guideline recommendations at pre-course (T0) and post-course (T1). Level of significance of observed differences in consumption in gram per day or per week for each food group between pre- to post-course as assessed by Wilcoxon signed-rank test, respectively: ns = not significant, *p* > 0.05; * *p* < 0.05; ** *p* < 0.01; *** *p* < 0.001.

It was assessed to what extent the medical students’ diet complied with the recommendations of the German Society for Nutrition. Most students’ food intake did not comply with those recommendations regarding fruits, vegetables, whole meal, dairy, fish, and meat. Thirty-one percent consumed enough fruits and 24% consumed enough vegetables. On the other hand, more than 80% of the students consumed red meat, processed meat, or fish maximum once a week. Among almost half of the students, the consumption of crisps and sweets exceeded the tolerated daily amount. In the group of students who participated in the lecture series, the proportion of those who followed the dietary recommendations with regard to the consumption of fruits, vegetables, nuts, dairy products, meat, and crisps and sweets significantly increased post-course (Figure 3). There was no significant difference in guideline adherence between the lecture participants and the comparison group at baseline, and no significant improvement in guideline adherence among the students in the comparison group was seen (Supplementary Table S5).

Furthermore, the proportion of lecture participants who described themselves as omnivorous decreased from 63% to 57%, while the proportions of vegetarians and vegans increased from 14.8% to 17.3% and from 12.7% to 14.6%, respectively (*p* < 0.001). Among the students in the comparison group, there was a slight decrease in the proportions of omnivores and vegans seen (omnivore: 54.7 % (T0) to 53.1 % (T1), vegetarian: 26.5 % (T0 + T1), and vegan: 7.8 % (T0) to 6.3 % (T1), *p* < 0.001).

### 3.3. Lifestyle Habits

**Figure 4:**
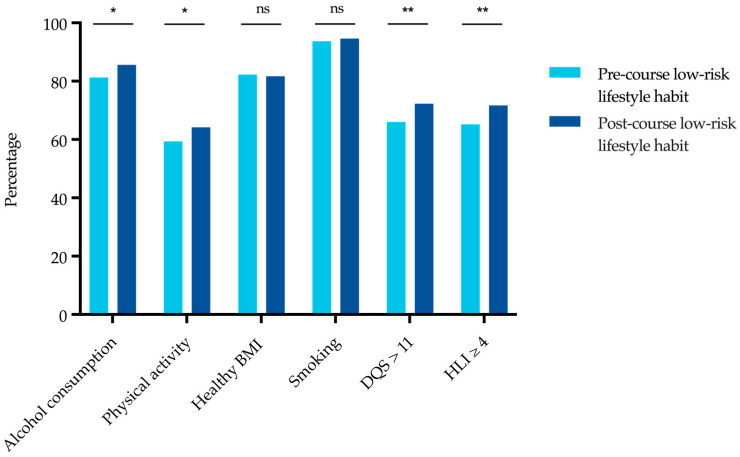
Percentage of lecture participants with low-risk lifestyle habits at pre- and post-course.Level of significance of observed differences between pre- to post-course as assessed by McNemar test, respectively: ns = not significant, *p* > 0.05; * *p* < 0.05; ** *p* < 0.01. Healthy BMI = BMI between 18.5 and 24.9 kg/m^2^; DQS = dietary quality score; HLI ≥ 4 = low-risk lifestyle habits in at least 4 of 5 lifestyle categories.

The students’ lifestyle habits were assessed with respect to alcohol consumption and physical activity. At baseline, one third of lecture participants stated that they drank alcohol rarely or never. After attending the lecture series, this proportion had further increased by 4% (*p* < 0.05). No such difference was seen in the comparison group (*p* = 0.18). Half of those who participated in the lecture series were physically active several times per week, while only 8% described themselves as inactive. Among the lecture participants, a statistically significant increase in physical activity was observed from pre- to post-course (*p* < 0.05), while the students in the comparison group appeared to be less physically active at T1 than they were at T0 (*p* = 0.549). A total of 82% of the students were of normal weight, and no significant difference in BMI was seen at T1 between the two groups. Smoking was found to be very uncommon in our cohort. Only 6.4 % of the lecture participants and 7.8% of the comparison group smoked at baseline. At post-course, significantly more students in the lecture participants had low-risk lifestyle habits in at least four out of five lifestyle categories (HLI ≥ 4) than at pre-course (65.2% at T0 vs. 71.7% at T1, *p* < 0.01) (Figure 4). At baseline, there was no significant difference in any lifestyle factors between the lecture participants and the comparison group. In addition, no significant changes were seen in the comparison group over time (HLI ≥ 4: 67.2% at T0 vs. 68.8% at T1, *p* = 1.0).

### 3.4. Medical Students Have a High Awareness of Healthy Nutrition and Planetary Health

**Figure 5:**
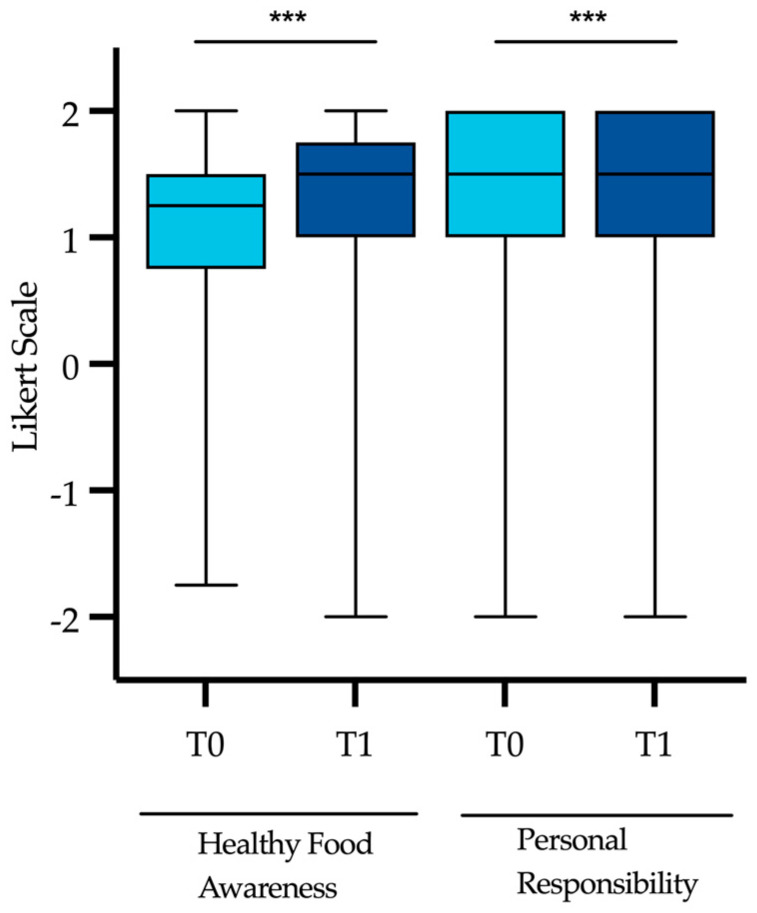
Comparison of median Healthy Food Awareness and Personal Responsibility among the lecture participants from pre- to post-course. Level of significance of observed differences between pre- and post-course as assessed by Wilcoxon signed-rank test, respectively: *** *p* < 0.001.

With regard to dietary behaviors (3.1–3.3), we analyzed the students’ attitudes toward healthy eating. Healthy Food Awareness among the medical students was already high initially with a median of 1.25, but it further increased to a median of 1.5 at post-course (*p* < 0.001) (Figure 5). From pre- to post-course, there was an improvement from 41.2% to 50.8% of students having a very high awareness of healthy nutrition (Healthy Food Awareness >1.5) (*p* < 0.001). In the comparison group, the median Healthy Food Awareness did not change significantly as it remained at 1.25 from T0 to T1 (*p* = 0.428).

With respect to planetary health, 66.3% of the participating students felt being personally responsible to adapt their individual nutritional habits to limit the progression of the climate crisis. After attending the lecture series, significantly more students had this feeling (74.8%, *p* < 0.001). In the comparison group, the median Personal Responsibility was 1.5 at T0 and T1 (*p* = 0.905), which was similar to the median among the lecture participants. Healthy Food Awareness and Personal Responsibility did not differ significantly between the lecture participants and the comparison group at baseline (*p* = 0.595, *p* = 0.855).

### 3.5. Determinants of Healthy Dietary Habits

In the multivariate linear regression analyses, it could be seen that, for the lecture participants at baseline, having an inactive life was significantly associated with a lower dietary quality score compared to the participants who indicated being more physically active (*p* < 0.001). In addition, the participants’ diet significantly influenced their DQS: being a vegetarian was associated with a lower DQS while adhering to a vegan or pescetarian diet was associated with a higher DQS when compared to following an omnivorous diet (*p* < 0.001, *p* < 0.05). Attitudes toward healthy eating influenced actual dietary behaviors, while Personal Responsibility did not show a significant association with the students’ DQS. Having a high Healthy Food Awareness, however, was associated with a better dietary quality (*p* < 0.001) (Supplementary Table S6).

## 4. Discussion

The main result of the study was that participating in the Germany-wide online lecture series “*Eat This!”*, which covered nutritional, public health, and planetary health aspects, most likely contributed to an improvement in the participants’ individual dietary guideline adherence as well as their Healthy Food Awareness and lifestyle habits. Furthermore, an increased awareness of Personal Responsibility regarding the role of individual behaviors in planetary health could be detected. Attending an online lecture series on nutritional medicine could, therefore, have beneficial effects on future physicians’ and their patients’ health.

### 4.1. Study Participants Have Healthier Dietary Habits and a Healthier Lifestyle than the General Population

Compared to the general population [36,37], the lecture participants had better dietary habits and a healthier lifestyle. These results are in line with other studies comparing the dietary habits of medical students to those of the national average [18]. In addition, the proportion of vegetarians and vegans in our study population was higher compared to the general population [38], but was similar to German university students from other subjects [39]. A reason for the better dietary habits and the higher proportion of vegetarians and vegans among the lecture participants compared to the general population could be the fact that healthier habits and vegetarians are found in higher socioeconomic strata [40], and medical students in Germany are likely to have a high socioeconomic status [41]. In addition, the high proportion of women among the participants could have had an influence as it has been shown that female students tend to have a better dietary quality [42]. Furthermore, there was a high proportion of participants who have strong feelings of personal responsibility to follow a plant-based diet to limit the progression of the climate crisis.

As Jacobs et al. showed in 2019, medical students in Germany have healthier dietary habits than the general population [41]. Nevertheless, poor adherence to recommendations on health-promoting behaviors is widespread among medical students worldwide, despite a supposedly positive influence of their studies [12,13,14,18,20]. Our study showed concurring results, with only 31% of the lecture participants consuming as many fruits and 24% consuming as many vegetables as recommended by the national guidelines. Possible explanations for the suboptimal dietary quality among students in general are the newfound freedom in the first years of study, which is a risk factor for poorer dietary behaviors [43,44], as well financial aspects as healthy and fresh food are often more expensive than energy-dense food [45]. In addition, the stress and workload among medical students are especially high [46], and education surrounding nutrition also seems to be inadequate [25]. Worldwide, the prevalence of smoking among medical students is 25% [47] even though awareness of the risks that tobacco has on their health should be higher. Night shifts and a heavy workload play a major role in explaining the unhealthy lifestyle among health professionals [47,48]. 

### 4.2. Attending “Eat This!” Probably Improved Guideline Adherence and Lifestyle Habits among Participants

Among the participants who attended the lecture series, the overall dietary quality slightly improved from pre- to post-course as indicated by the increased DQS among the lecture participants. This did not increase in the comparison group. Furthermore, a better guideline adherence among the lecture participants was found for 7 out of 10 food groups after attending the lecture series, and individual lifestyle habits, such as physical activity, improved among the lecture participants, but not in the comparison group. Meanwhile, alcohol consumption also decreased in the comparison group, perhaps suggesting that a difference in alcohol consumption might rather be influenced by external confounders, such as the COVID-19 pandemic or seasonal fluctuations [49,50]. Other studies also showed an improvement in nutritional quality assessed as changes in dietary habits among college students participating in lectures on basic nutritional knowledge [51,52,53,54].

Among medical students, improvements in dietary habits and personal lifestyle, a higher awareness of dietary choices, and an increased frequency of nutritional counseling after attending a course on nutritional medicine had been reported [12,21]. In contrast to our nationwide online lecture series which was voluntary and could be attended by medical students from almost all faculties in Germany, Conroy et al. and Crowley et al. assessed the impact of teaching interventions that were already part of the medical curriculum. The intervention by Conroy et al. included 14 weekly sessions with a mix of lectures and problem-based and case-based learning. In the intervention by Crowley et al., nutritional medicine content was included in a 12-week general course on the digestive system. Even though our lecture series was offered exclusively online and had a lower contact time than those previously mentioned, it was found that participation in the lecture series might have positively influenced the individual dietary choices of the participating medical students, too.

### 4.3. Healthy Food Awareness, Physical Activity, and Following a Vegan or Pescetarian Diet Are Related to a Better Dietary Quality

The participants already had a high Healthy Food Awareness at pre-course, which further increased from pre- to post-course and indicated the linkage of the perceived importance of healthy eating to individual health and personal well-being. Interest in nutrition was likely to be high among the study participants as participation in the lecture series was voluntary. The improvement in dietary habits and lifestyle and the increase in Healthy Food Awareness might be due to an increase in knowledge as a result of the lecture series, as suggested by the data from Mota et al. [18]. However, the high proportion of vegetarians in our study must be taken into account, as vegetarians could be more easily influenced with regard to a change in their awareness and diet due to their preconceptions.

Having a high Healthy Food Awareness was associated with a better dietary quality. Furthermore, being more physically active and following a vegan or pescetarian diet showed positive associations with the DQS. This might provide further evidence of the link between a higher awareness of a healthy lifestyle and corresponding healthier eating behaviors, especially at post-course. Following a vegetarian diet, however, was associated with a lower DQS compared to following an omnivore diet. One explanation for this could be that the DQS calculator placed a lot of emphasis on fish and sugar. If the vegetarian students did not eat fish but ate a lot of sugary foods, the DQS was especially poor. To assess the overall diet quality, other FFQs and scores should be used in the future to further explore the nutritional quality of different diets among medical students.

Nonetheless, the lecture series may contribute to personal health by improving medical students’ dietary habits and lifestyle and raising awareness of healthy nutrition, which in turn may have positive effects on medical students’ dietary behaviors [23,55].

### 4.4. Attending the Lecture Series Contributes to Nutritional Education for Medical Students and Could Lead to Future Doctors Providing More Counseling on Nutrition and Planetary Health

Calls to improve nutritional education for physicians and increase the perceived importance of healthy lifestyles among physicians have been made for many years [56,57]. On the one hand, a healthy lifestyle has a personal benefit in reducing all-cause mortality and the risk of developing an NCD. On the other hand, improved lifestyle and a balanced, plant-based diet contribute to the mitigation of the climate crisis. Therefore, it is essential that physicians practice more nutritional and lifestyle counseling to tackle the global burden of disease [9]. Since the individual lifestyle of physicians probably has an impact on the frequency of nutritional counseling [23,55], nutritional curriculum initiatives may increase the frequency of nutritional counseling by influencing future physicians’ dietary habits and attitudes, thus possibly improving the prevention and treatment of NCDs [23,55,58].

A teaching event, such as *“Eat This!”*, may be suitable to improve the dietary behaviors and lifestyle of medical students, which may lead to further improvements in individual well-being and enhance the personal and professional success of future physicians; studies have shown that healthier lifestyles, high self-efficacy, and self-perceived competence lead to increased well-being [59,60,61]. Another advantage of increasing education in nutritional medicine could be the possible resulting increase in nutritional counseling. On the one hand, physicians could, if adequately trained, counteract a further increase in the prevalence of NCDs and reduce health care costs. On the other hand, healthy lifestyles can also be more climate-friendly. Healthy nutrition, in many ways, correlates with climate-friendly nutrition [1]. Furthermore, other risk factors for NCDs, such as an inactive lifestyle, are linked to climate-damaging behaviors and a shift to a more sustainable lifestyle can avoid up to 30% of individual greenhouse gas emissions [9,62].

### 4.5. Strengths and Limitations

The percentage of female study participants was 10% higher than the proportion of female medical students in Germany in general [63]. This may be due to the higher interest in nutritional medicine topics among women. In previous comparable studies, more women than men participated [64]. In general, a selection bias for participating in our study cannot be excluded. Since the study was conducted using a convenience sample, it could be possible that it is not representative of all medical students. Yet, there were no statistically significant differences in dietary behaviors between the lecture participants and the comparison group.

However, the lower number of participants in the comparison group compared to the group of lecture participants should be taken into account when interpreting the results. All analyses including the comparison group are considered exploratory.

Although the questionnaire was not validated as a whole, questions from a validated short-form FFQ were used to enable the best possible measurement quality for our outcomes. A strength of our study was the large number of participants compared to other studies on the dietary behaviors of students [12,18,20,52,53].

One might speculate that some of the students abstained from animal products in January, as the “Veganuary” initiative annually calls for following a vegan diet during this month. However, there was no increase in vegan students in the comparison group. Again, one has to keep in mind that the results do not prove causality and that the characteristics of the sample, such as the high percentage of women and vegetarians and their interest in nutritional initiatives, might have been particularly high among the lecture participants, which might have contributed to the behavioral changes observed.

## 5. Conclusions and Outlook

Although one might expect a stronger influence of medical studies on individual dietary habits, non-adherence to national recommendations on healthy eating is quite common among medical students.

Our study shows, however, that a low-threshold offer, such as the online lecture series *“Eat This!”*, can positively influence eating behaviors, lifestyles, and Healthy Food Awareness among medical students. The results indicate that including nutritional medicine into medical education may be a promising way to improve future physicians’ and their patients’ dietary habits. This has the potential to increase the likelihood that participants will practice more nutritional and behavioral change counseling as future physicians, thereby also fulfilling their role in combating the global burden of NCDs and mitigating the climate crisis.

Future investigations with a randomized controlled study design and a bigger comparison group should be carried out to confirm our findings. In addition, further studies including follow-ups should compare the effectiveness of different teaching and learning formats and investigate the long-term effects of educational interventions on nutritional medicine.

## Figures and Tables

**Table 1:** Demographics and lifestyle parameters of participants included in the longitudinal analyses at baseline (T0).

		Lecture Participants *n* = 520	Comparison Group *n* = 64	*p*-Value
Sex [*n* (%)]				0.078
	male	106 (20.4)	14 (21.9)	
	female	414 (79.6)	50 (78.1)	
Age ^a^	Years	23 (22–26)	24 (23–26)	0.084
Study time ^a^	Semester	7 (5–9)	8 (5–11)	0.061
Study phase [*n* (%)]				0.896
	Preclinical	111 (21.3)	15 (23.4)	
	Clinical	383 (73.7)	42 (65.6)	
	Practical Year	26 (5.0)	7 (10.9)	
BMI ^a^	kg/m^2^	21.5 (20–23.3)	21.5 (20.2–23.8)	0.136
Physical activity per week ^a^		3 (2–4)	3 (2–4.75)	0.381
Smoking [*n* (%)]				0.365
	Yes	33 (6.4)	5 (7.8)	
	No	487 (93.7)	59 (92.2)	
	n/a	0 (0)	0 (0)	
Alcohol consumption [*n* (%)]				0.545
	Low	309 (59.4)	37 (57.8)	
	Medium	197 (37.9)	25 (39.1)	
	High	13 (2.5)	2 (3.1)	
	n/a	1 (0.2)	0 (0)	
Diet [*n* (%)]				0.913
	Omnivore	328 (63.1)	35 (54.7)	
	Vegetarian	77 (14.8)	17 (26.5)	
	Vegan	66 (12.7)	5 (7.8)	
	Pescetarian	35 (6.7)	4 (6.3)	
	Other	14 (2.7)	3 (4.7)	
	n/a	0 (0)	0 (0)	
DQS ^b^		10.8 ± 1.35	10.67 ± 1.04	0.430
HLI ≥ 4 [*n* (%)]		339 (65.2)	43 (67.2)	0.838

^a^: Median (interquartile range), ^b^: Mean ± standard deviation. Alcohol consumption categories: Low = alcohol consumption less than once per week; Medium = consumption one to three times per week; and High = consumption more than 4 times per week. BMI = Body Mass Index. DQS = dietary quality score. HLI = Healthy Lifestyle Index.

## Data Availability

The data presented in this study are available from the corresponding author upon request. The data are not publicly available as they contain information that could compromise the privacy of research participants.

## Supplementary Materials

The following supporting information can be downloaded at https://www.mdpi.com/article/10.3390/nu15030580/s1, Table S1: Lecture topics; Table S2: Items per food group and criteria for the guideline adherence score; Table S3: Calculation of consumption frequencies per 28 days per answer category; Table S4: Standard amount in gram per serving size; Table S5: Food intake in relation to the national guideline recommendations at pre- and post-course among lecture participants and students in the comparison group in percentage; Table S6: Multivariate linear regression analysis: Associations of lecture participants’ characteristics, lifestyle habits, and attitudes with the dietary quality score at baseline (*n* = 1224).
