# Supplementary material for: Improvement of Dietary Habits among German Medical Students by Attending a Nationwide Online Lecture Series on Nutrition and Planetary Health (“Eat This!”)
Source: Nutrients. 2023 Jan 22;15(3):580. doi: 10.3390/nu15030580 (PMC9920441; doi:10.3390/nu15030580)
Supplement: Supplementary file 1 [file nutrients-15-00580-s001.zip › nutrients-2078172-supplementary.pdf]

Supplementary Material

Improvement of dietary habits among German medical students by attending a nation-wide online lecture series on nutrition and planetary health *("Eat This!")*.

Anna Helbach, Moritz Dumm, Katharina Moll, Tim Böttrich, Can Gero Leineweber, Wiebke Müller, Jan Matthes†, Maria Cristina Polidori†

† Equal contribution.

Supplementary Table S1: Lecture schedule of the lecture series "*Eat This*!" from November 2020 - January 2021

	Title	Contents
1	Introduction to Nutritional Medicine	The importance of nutrition as a risk factor for non-communicable diseases
2	Nutritional Psychology and behavior change	Behavior change counseling, motivational interviewing, essential models of nutritional psychology, satiety and appetite regulation, reward system
3	Nutritional Medicine and Gastroenterology	Definitions of microbiome, prebiotics, probiotics, gut-brain axis, gut dysbiosis and correlations with diseases, intolerances (gluten, fructose, lactose) and allergies
4	Nutritional therapy in Oncology and Pharmaconutrition	Tumor cachexia, nutritional assessment in oncology patients, nutritional risk factors for cancer, pharmaconutrition (drug-nutrient interactions)
5	Nutritional Medicine and Geriatrics	Definitions (sarcopenia), nutritional risk factors for dementia, risk factors and consequences of malnutrition, indications and characteristics for artificial nutrition (enteral, parenteral)
6	Nutrition and Climate Change	Role of nutrition in the climate crisis and mitigation of global warming, role of nutrition and agriculture in the development of antibiotic resistance and global pandemics
7	Nutritional Medicine in Cardiology	Lipoprotein Metabolism and Pathogenesis of Cardiovascular Disease, Nutritional Recommendations for Patients (Fatty Acids, Salt, DASH Diet)
8	Nutritional therapy of autoimmune diseases and fasting	Definitions, benefits of including and excluding individual food groups, types of fasting, potential health benefits and risks of fasting
9	Plant-based diets in childhood and Nutrition in Pediatrics	Definitions, advantages and disadvantages of vegan diets, critical nutrients, supplementation of critical nutrients in childhood, prognostic relevance of childhood obesity, childhood malnutrition
10	Nutritional Medicine in Diabetology	Definition of BMI, obesity, metabolic syndrome and forms of diabetes, pathophysiology of diabetes, nutritional recommendations for diabetic patients and treatment options
11	Nutrition and Public Health	Health systems, health care financing, current threats to public health, food pricing and taxation, food supply

Supplementary Table S2. Items per food group and criteria for the guideline adherence score.

Foodgroup	Items	Criteria for a score of 0 (below guideline recommendation*)	Criteria for a score of 1 (meeting guideline recommendation*)	Criteria for a score of 2 (Above guideline recommendation*)
Fruits	Fruits, Fruitjuice max. 1/day	<250 g / day	>250 g / day	Not applicable
Vegetables	Vegetables, Salad, Legumes	<400 g / day	>400 g / day	Not applicable
Nuts	Nuts	0 g / day	1-25 g / day	>25 g / day
Wholemeal	Fibre-rich cereals, porridge, oats, Muesli, wholemeal bread	<200 g / day	200-360 g / day	>360 g / day
Dairy	Cheese, Yoghurt, Milk	<250 g / day	250-300 g / day	>300 g / day
Fish	Fish (all forms) and Seafood	<150 g / week	150-220 g / week	>220 g / week
Meat	Red meat, white meat, processed meat	<300 g / week	300-600 g / week	>600 g / week
		Criteria for a score of 0 (above max. tolerated amount)	Criteria for a score of 1 (below max. tolerated amount)	
Eggs	Egg	>180 g / week	<180 g / week	
Sweet beverages	Fruit Juice, Icetea, Softdrinks	>192 g / day	<192 g / day	
Crisps and sweets	Crisps, Salty Snacks, Sweets, Cake, Chocolate, Ice Cream	>49 g / day	<49 g / day	

*Recommendations as indicated by the German Nutrition Society[6] and Kuhn[30].

Supplementary Table S3. Calculation of consumption frequencies per 28 days per answer category.

Answer categories	Frequency	Amount per 28 days	Calculation
0	Rarely/never	0	0*0
1	Less then once a Week	1	1*1
2	Once a Week	4	1*4
3	2-3 times a Week	10	1.5*4
4	4-6 times a Week	20	5*4
5	1-2 times a Day	42	1.5*28
6	3-4 times a Day	98	3.5*28
7	5+ times a Day	168	6*26
	Meat		
0	Rarely/never	0	0*0
1	Less then once a Week	1	1*1
2	Once a Week	4	1*4
3	2-3 times a Week	10	1.5*4
4	4-6 times a Week	20	5*4
5	7+ a Week	42	1.5*28

Calculation adapted from Kuhn[30].

Supplementary Table S4. Standard amount in gram per serving size.

Food item	Standard serving size	Standard amount in g
Fruit	1 Piece	150
Fruitjuice	1 Glass (200ml)	200
Salad	1 Serving	150
Vegetables	1 Serving	150
Fried potatoes	1 Serving	150
Legumes	1 Serving	150
Cereals	1 Serving	50
Wholemeal bread	1 Slice	50
Eggs	1 Egg	60
Milk	1 Serving	50
Cheese / Yoghurt	1 Serving	150
Nuts	1 Serving	25
Crisps, Cracker	1 Serving	50
Sweets, Cake	1 Serving	100
Ice Cream	1 Serving	75
Softdrinks	1 Glass (200ml)	200
Red meat	1 Serving	120
Poultry	1 Serving	150
Processed meat	1 Serving	150
Fried white fish	1 Serving	90
White fish	1 Serving	90
Oily fish	1 Serving	90

Standard amounts per serving size adapted from German Nutrition Society[6] Kuhn[30].

Supplementary Table S5. Food intake in relation to national guideline recommendations pre- and post-course of lecture participants' as well as of students in the comparison group in percent.

	T0			T1			
Foodgroup	Below Recomm-endation %	Meeting Recomm-endation %	Above Recomm-endation %	Below Recomm-endation %	Meeting Recomm-endation %	Above Recomm-endation %	p-value
Lecture participants							
Fruits*	68.8	31.2	-	66.5	33.5	-	0.011
Vegetables***	75.6	24.4	-	69	31	-	<0.001
Nuts***	20.6	57.5	21.9	15.8	52.1	32.1	<0.001
Wholemeal	97.5	1.9	0.6	97.7	1.5	0.8	0.250
Dairy*	77.3	15.8	6.9	81	12.8	6.2	0.004
Fish	89.6	4.8	5.6	87.3	5.8	6.9	0.059
Meat***	70.6	19.6	9.8	78.1	14.6	7.3	<0.001
Sweet beverages	-	95	5	-	94.6	5.4	0.011
Crisps and sweets**	-	54.4	45.6	-	60.4	39.6	0.002
Eggs	-	75.6	24.4	-	77.7	22.3	0.250
Comparison Group							
Fruits	65.6	34.4	-	68.8	31.2	-	0.65
Vegetables	76.6	23.4	-	81.3	18.7	-	0.196
Nuts	28.1	53.1	18.8	29.7	54.7	15.6	0.302
Wholemeal	98.4	0	1.6	95.3	3.1	1.6	0.418
Dairy	78.1	18.8	3.1	81.3	12.5	6.2	0.194
Fish and Seafood	92.2	4.7	3.1	90.6	3.1	6.3	0.161
Meat	84.4	7.8	7.8	78.1	18.8	3.1	0.644
Sweet beverages	-	90.6	9.4	-	96.9	3.1	0.557
Crisps and sweets	-	54.7	45.3	-	46.9	53.1	0.475
Eggs	-	82.8	17.2	-	82.8	17.2	0.444

Level of significance of observed differences in consumption in gram per day or per week for each food group between pre- to post course as assessed by Wilcoxon signed-rank test, respectively: ns = not significant, $p > 0.05$, * $p < 0.05$, ** $p < 0.01$, *** $p < 0.001$.

Supplementary Table S6. Multivariate linear regression analysis: Associations of lecture participants' characteristics, lifestyle habits and attitude with the dietary quality score at baseline (*n* = 1224).

Independent Covariate		Regression coefficient B	CI 95%		Std. Error	*p*
Age	< 20 (Ref)					
	20-24.9	-0,278	-0,728	0,172	0,229	0,226
	≥25	-0,441	-0,905	0,023	0,236	0,063
Sex	Male (Ref)					
	Female	-0,096	-0,28	0,088	0,094	0,305
BMI categories	Normal weight (Ref)					
	Underweight (<18.5 kg/m^2)	-0,11	-0,379	0,158	0,137	0,419
	Overweight / obese (≥25 kg/m^2)	0,135	-0,098	0,368	0,119	0,256
Alcohol consumption	None or moderate (Ref)					
	High	0,079	-0,099	0,257	0,091	0,385
Physical activity*	Active (Ref)					
	Inactive	-0,294	-0,445	-0,143	0,077	<0.001
Smoking	No (Ref)					
	Yes	0,187	-0,057	0,431	0,124	0,133
Diet*	Omnivore (Ref)					
	Vegetarian	-0,236	-0,428	-0,043	0,098	0,017
	Vegan	0,399	0,169	0,628	0,117	<0,001
	Pescetarian	0,313	0,043	0,583	0,138	0,023
Study phase	Preclinical (Ref)					
	Clinical	0,156	-0,025	0,336	0,092	0,091
	Practical year	-0,069	-0,359	0,222	0,148	0,643
Healthy Food Awareness*	High (Ref)					
	Low	-0,548	-0,702	-0,394	0,079	<0.001
Personal Responsibility	High (Ref)					
	Low	-0,05	-0,21	0,109	0,081	0,534

Model's goodness of fit (R^2) = 0.112

**p*-value < 0.05 is considered as statistically significant. *n* = 1224.
